# Recurrent Flooding and Household Food Access in Central Java, Indonesia

**DOI:** 10.3390/ijerph21101370

**Published:** 2024-10-17

**Authors:** Breanne K. Langlois, Aris Ismanto, Leah Beaulac, Katherine Berry, Magaly Koch, Timothy Griffin, Erin Coughlan de Perez, Elena N. Naumova

**Affiliations:** 1 Friedman School of Nutrition Science and Policy, Tufts University, Boston, MA 02111, USAtimothy.griffin@tufts.edu (T.G.); erin.coughlan@tufts.edu (E.C.d.P.); elena.naumova@tufts.edu (E.N.N.); 2 Department of Oceanography, Faculty of Fisheries and Marine Science, Universitas Diponegoro, Semarang 50275, Indonesia; aris109@lecturer.undip.ac.id; 3 Center for Remote Sensing, Boston University, Boston, MA 02215, USA; mkoch@bu.edu; 4 Red Cross Red Crescent Climate Centre, 2502 KC The Hague, The Netherlands

**Keywords:** flood, Indonesia, natural disasters, extreme events, data usability, food security, nutrition security, food access, diet diversity, climate and environmental change

## Abstract

It is unknown how recurring flooding impacts household diet in Central Java. We aimed to assess how recurrent flooding influenced household food access over 22 years in Central Java by linking the Global Surface Water dataset (GSW) to the Indonesian Family Life Survey. We examined linear and nonlinear relationships and joint effects with indicators of adaptive capacity. We measured recurrent flooding as the fraction of district raster cells with episodic flooding from 1984–2015 using GSW. Food access outcomes were household food expenditure share (FES) and dietary diversity score (DDS). We fit generalized linear mixed models and random forest regression models. We detected joint effects with flooding and adaptive capacity. Wealth and access to credit were associated with improved FES and DDS. The effect of wealth on FES was stronger in households in more flood-affected districts, while access to credit was associated with reduced odds of DDS in more flood-affected districts. Flooding had more predictive importance for FES than for DDS. Access to credit, a factor that ordinarily improves food access, may not be effective in flood-prone areas. Wealthier households may be better able to adapt in terms of food access. Future research should incorporate land use data to understand how different locales are affected and further understand the complexity of these relationships.

## 1. Introduction

It is known that climate events are impacting food security around the world through direct losses to agricultural crops, presenting a major challenge to food and nutrition security through many pathways [1,2,3,4,5]. In the 6th Assessment Report, the Intergovernmental Panel on Climate Change (IPCC) estimates increased nutrition-related diseases and undernourishment because of ongoing climate changes [5]. Flooding, caused by significant variations in rainfall patterns and other factors, is threatening our food system [1,2]. The FAO reports that drought and flood are the greatest culprits of agricultural production loss and estimates that these production losses lead to loss of calories and nutrients [2]. The impacts to household diet and nutrition, however, are not well understood. While increased rainfall has been shown to have a positive effect on diet diversity in some regions, the current evidence base for flooding is poor [3,6].

Much of the research on flooding and food security has focused on food production or availability, leaving out the other diet-related dimensions at the household level [1,7]. Some studies have looked at diet diversity following floods [8,9,10,11]. Some evidence of undernutrition at the household level suggests a link between extreme floods and stunting [12,13,14,15]. The sparseness of these studies and their heterogeneity make causal inference difficult. While flood impacts on disease outcomes are better understood, more high-quality interdisciplinary studies are needed to understand the impacts to the multiple dimensions of food and nutrition security, diet, and nutritional status [16,17,18].

Furthermore, many prior studies have focused only on single flood events, while the cumulative effects of recurrent flooding over time are understudied [8,9,10,11,15,19,20]. This is largely due to lack of integration of spatial and epidemiological datasets. With an abundance of climate data in the public domain spanning decades, there is an opportunity to create integrated datasets to study the human impacts of climate events.

Central Java, Indonesia, is an important case study of how recurrent flooding impacts household-level food security. Due to a confluence of geological, environmental, and economic factors, Central Java faces recurrent fluvial and tidal flooding from tropical storms, intense rainfall, sea level rise, and land subsidence [21,22]. In 2023, Central Java was among the most flood-affected provinces in Indonesia, totaling about 93 events according to the National Disaster Management Agency (BNPB) [23]. Central Java is also predominantly agricultural, producing most of Indonesia’s rice supply [24]. Another important aspect is the proclivity to stay and adapt in response to floods [25,26,27,28]. While this area is highly vulnerable to flood hazards, studies related to food security in this context have mainly focused on crop production [29,30,31,32,33]. The impacts of flooding on household diet and nutrition in this area are not fully understood.

In this paper, we address three gaps: 1. lack of datasets that integrate information about the environment and human outcomes over time; 2. limited understanding of the impact of flooding on household diet and nutrition; and 3. limited understanding of the impacts of recurring floods. We address the data gap by linking the Global Surface Water (GSW) dataset to the Indonesian Family Life Survey (IFLS) [34,35]. The GSW describes the long-term spatiotemporal distribution of surface water and its changes. The IFLS provides data on 1949 Central Java households over 22 years from 1993 to 2015 [7,34]. The integration of these datasets allows us to explore questions that were previously not possible. Here, we examine the relationship between recurring flooding and household access to sufficient quantities of food and adequate nutrition in Central Java.

We explore the form and shape of the relationship by examining nonlinearity in our models and differential effects with indicators of adaptive capacity. As flooding and climate-related hazards are expected to worsen, there is a need to understand the impacts on diet and nutrition to identify key areas for intervention to achieve sustainable development goals number 2 (Zero Hunger) and 13 (Climate Action). Further, a more comprehensive understanding of the impacts of disasters, including flooding, on food and nutrition security is needed for climate resilience policies [1]. With no universal definition or metric of flood recurrence, the ability to compare study findings within and across contexts is limited, impacting research-informed policy recommendations. This study adds to this knowledge gap by exploring a metric of flood recurrence using existing data.

## 2. Materials and Methods

### 2.1. Data Sources and Survey Design

We conducted a longitudinal study of Central Java households using all 5 waves of IFLS. The IFLS is a nationally representative, longitudinal survey designed to study various aspects of social and economic well-being of the Indonesian population. Our dataset included modules from Books K (the control book), 1, and 2 of the household survey: household characteristics (section KRK), consumption and expenditures (section KS), farm and non-farm business (sections UT and NT), household assets (section HR), and household economic shocks (section GE). The first wave of IFLS was conducted in 1993-94. The following 4 waves were conducted in 1997–1998, 2000, 2007–2008, and 2014–2015, respectively.

The IFLS was conducted by the RAND Corporation [34]. All waves underwent ethical approval by the RAND Institutional Review Board (IRB) in the United States and the University of Gadjah Mada IRB in Indonesia prior to the collection of any data. The approval number given by the RAND IRB is s0064-06-01-CR01.

Sampling for all waves was based on Wave 1 (1993–1994), which used 2-stage stratification of province and urban/rural location. Provinces were selected to capture the diversity of the population to maximize representativeness. For cost reasons, only 13 of the 27 provinces were sampled in 1993. In Wave 1, the goal was to achieve a final sample size of 7000 households. Assuming 10% nonresponse, the sample size was inflated to 7730. A total of 7224 households completed interviews in Wave 1. This represented ~83% of the 1993 population.

IFLS had an excellent tracking rate. For Waves 2–5, the survey attempted to reinterview “origin” households, i.e., households from Wave 1 that contained at least 1 member of the Wave 1 interviewed household. If a household had moved, the survey attempted to locate them. The survey also contacted “split-off” households. These were households containing a target member of an original household (i.e., a Wave 1 household member that was an individual panel respondent or was over the age of 26 or other criteria). In Waves 2 through 5, a total of *n* = 6821, *n* = 6661, *n* = 6596, and *n* = 6432 of the 7224 origin households, respectively, were recontacted. We analyzed the sample of 2265 Central Java households (origin and split-off) included across all waves.

The GSW measures long-term changes in surface water, globally, from Landsat satellite imagery gathered over decades [36]. GSW is a collection of datasets produced by the European Commission’s Joint Research Centre under the Copernicus Program for a broad array of uses, including water resource management, conservation, and food security. The GSW collection includes datasets of surface water occurrence, change, seasonality, recurrence, transitions, and maximum extent. The long timespan (over 3 decades), use of millions of satellite images, and 30 m pixel resolution make GSW data products of high quality.

We used version 1 of GSW covering the timespan from 1984 to 2015, as it corresponded with the IFLS study period. GSW datasets do not vary by time. Rather, they capture the total changes throughout the period. We used the “Recurrence” dataset to estimate the degree of recurrent flooding over the timespan. This variable captures inter-annual change, describing the frequency with which water returned from year to year. A detailed description of GSW variables is available in the associated paper [36].

### 2.2. Dataset Preparation

We created a multilevel dataset, structured by household wave. We subset it to Central Java, including all Central Java households regardless of their time in the study (i.e., whether they appeared in 1 or multiple waves). The final dataset contained 6743 observations, 2265 unique households from Central Java across 5 survey waves.

To integrate spatial imagery (raster) data from GSW with IFLS, we needed to summarize the pixel values by district—the smallest administrative boundary in the IFLS. We accomplished this through the following steps. First, we downloaded GSW “recurrence” data as GeoTIFF (raster) files. Next, we obtained vector (polygon) data of Indonesian subnational administrative boundaries from the Humanitarian Data Exchange. We then used R Statistical Software (v4.2.2; R Core Team 2022) to obtain the district-level summaries of GSW raster data [37]. We achieved this by cropping and masking the raster to Central Java, then extracting and summarizing the raster values by the district polygons using the “exactextractr” package. This produced a dataset of summary statistics for each Central Java district. Summary statistics included the sum of raster cells covered by each district and the fractions of cells corresponding to each value within the district. Finally, the results were merged to IFLS by district. Since the GSW raster data captured the change throughout the timespan and did not vary by time, we performed a “one-to-many” merge.

### 2.3. Variables

We approximated *recurrent flooding* using the “recurrence” layer from GSW covering the period from 1984 to 2015. This variable was defined as the fraction of district raster cells with episodic flooding over the period (GSW recurrence values between 1 and 99). GSW recurrence ranges from 0 to 100 discrete values where 0 is not water, 1 is 1% recurrence over the period, and 100 is 100% recurrence. Values of 100 represent seasonal or permanent water bodies, while values from 1 to 99 represent a range of episodic flooding. By summing the fractions of the districts with recurrence values between 1 and 99, we obtained a variable representing the total portion of the district that had episodic (i.e., nonseasonal or unpredictable) flooding over the period. We assume that districts with a larger portion of episodic flooding over the period were affected by more floods. This metric is exploratory, as similar metrics from which to base our definition of recurrent flooding are lacking.

Our outcomes were constructed from household consumption and expenditure data and based on the Data4Diets framework [7]. We used household food expenditure share to measure diet quantity, expressed as the percentage of total spending spent on food, calculated by dividing the total household expenditure spent on food by the total household expenditure, multiplied by 100. It has been well documented that the share of income spent on food increases with a household’s vulnerability, known as Engel’s law [7]. Accordingly, with rising income, nonfood expenditure increases more than food expenditure, thus decreasing food expenditure share [7,38].

We used household dietary diversity score to measure diet quality, expressed as an integer from 0 to 12 (low to high). This variable was calculated as the total number of food groups consumed by the household in the past week as follows: cereals, roots and tubers, vegetables, fruits, meat/poultry/offal, eggs, fish and seafood, pulses/legumes/nuts, milk and milk products, oils/fats, sugar/honey, and miscellaneous. This selection of food groups is based on prior work by Food and Nutrition Technical Assistance (FANTA) [39]. We constructed the variable using data from the household expenditures and consumption section in Book 1 (section KS), which contained the types of foods (a 37-item list) reported by the household. We generated indicator variables corresponding to each of the 12 food groups listed above from the types of food items (e.g., rice and corn were categorized as cereal; sago, cassava, tapioca, dried cassava, and other staple foods, like sweet potatoes, potatoes, and yams, were categorized as roots and tubers). The food groups were then summed to create the integer variable. This serves as a useful indicator of diet quality, as it is known that a more diverse diet is associated with a range of positive outcomes, including improved anthropometry, caloric and protein adequacy, and income [7,39]. Together, both indicators serve as proxies of household food access, measuring improved household food consumption [39].

**Figure 1:**
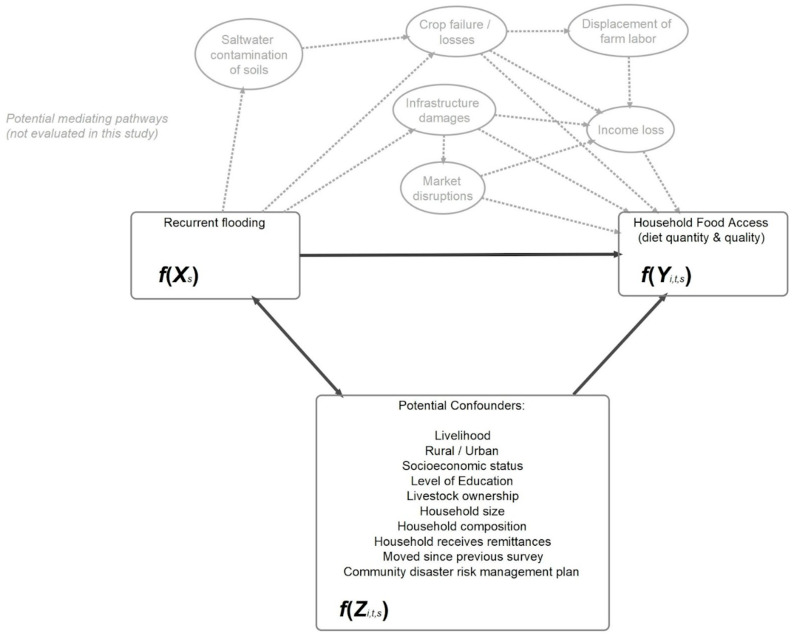
Conceptual framework.

Our selection of covariates was based on a conceptual framework from prior evidence relating these factors to food security (Figure 1) [3,7,39]. These factors may also be determinants of living in more heavily flooded areas. We were unable to include a variable indicating whether the community had a disaster risk management plan as we did not have the relevant data. We did not include level of education due to a high percentage (>20%) of missing values.

### 2.4. Modeling

We took a hybrid approach to our modeling. Our main interpretation is taken from statistical models to explain the relationships in our data. However, we also used a machine learning method—random forest algorithm—to assess predictive accuracy and add context to our statistical findings.

We used Stata 17 (Stata Statistical Software: College Station, TX, USA: Stata Corp LP) for all analyses. We constructed a wealth index to measure socioeconomic status using principal component analysis, based on a methods guidance paper from the World Food Programme [40]. We checked assumptions using Bartlett’s test for sphericity and the Kaiser–Meyer–Olkin Measure of Sampling Adequacy. Variables with low factor loadings or that were related in the opposite direction were removed. A listing of the variables that were included in the wealth index are shown in the descriptive table (Table 1) in the results section.

Our dataset contained 3 levels of hierarchy: observations made at each cross-sectional survey wave (level 1), for each household (level 2), and within each district (level 3). We included two random effects in our statistical models to account for the correlation by household and district, described by the following notation:

$$f\left(Y_{itsv}\right)=\beta_{0}+\beta_{1}\left({f\left(X\right)}_{s}\right)+\beta_{2}\left({f\left(Z\right)}_{itsu}\right)+\beta_{3}K_{i}+b_{s}+b_{is}+\varepsilon_{its}$$

In the above equation, $f(Y_{itsv})f(Y_{itsv})$ represents a function of the outcome *v* for household *i* at time *t* within district *s*; $\beta_{1}\left({f\left(X\right)}_{s}\right)\beta_{1}\left({f\left(X\right)}_{s}\right)$ represents the coefficient for a function of the flood variable within district *s*; $\beta_{2}({f(Z)}_{itsu})\beta_{2}({f(Z)}_{itsu})$ represents the coefficient for a function of the confounding factors, *u*, displayed in Figure 1 for household *i* at time *t* within district *s*. $\beta_{3}K_{i}\beta_{3}K_{i}$ represents the coefficient for the survey wave for household *i*. The random effects $b_{s}b_{s}$ and $b_{is}b_{is}$ vary across districts and households within districts, respectively.

We fit linear mixed regression models and examined specification errors using the link test, multicollinearity of independent variables using variance inflation factor, and residual plots. We checked for outliers using Cook’s distance after fixed-effect models. For the dietary diversity score outcome, there was a specification error and evidence of heteroscedasticity. We first tried transformations of the dependent variable, which improved but did not remedy the heteroscedasticity. Therefore, we present ordered logistic mixed regression for the dietary diversity score outcome. We present a proportional odds model with mixed effects, although this assumption was not met for all variables. We explored nonlinearity using a purely functional approach by fitting linear and quadratic terms of our continuous flood variable. We used likelihood ratio tests to determine the added value of the quadratic terms. We conducted complete case analysis as there were little missing data (<5%).

We used the following indicators to assess adaptive capacity, which were only available in Waves 3 to 5: knowledge of where to borrow money, secured a loan in the past 12 months, received any assistance in the past 12 months, and received cash assistance. We examined their joint effects with flooding in separate models.

We conducted random forest regression models for both outcomes. To determine the optimal number of iterations to include in the model, we examined scatter plots of out-of-bag error versus varying number of iterations. For both models, error converged at ~400 iterations. We present results in variable importance plots.

## 3. Results

Of 2265 Central Java households, *n* = 856 were sampled in Wave 1, *n* = 998 in Wave 2, *n* = 1325 in Wave 3, *n* = 1616 in Wave 4, and *n* = 1948 in Wave 5. The tracking rate was 97% (*n* = 827 of 856 origin households in Wave 5). The rest of the sample was “split-off” households (new households containing a target member of an original household). The sample can be characterized as about half rural, a third agricultural, mostly homeowners, living with moderate-sized yards and improved water sources and facilities (Table 1).

**Table 1:** Descriptive characteristics of Central Java households in their first wave of the survey ^1^.

Characteristic	Variable	First Wave
Sample size		2265
Livelihood	Has land for farming ^3^	269 (22%)
	Anyone was a farmer in the past year	755 (34%)
	Anyone engaged in fishing (non-fishery) ^3^	10 (9%)
Livestock ownership	Owns livestock/poultry/fishpond	619 (27%)
Highest level of education ^2^	Elementary	507 (35%)
	Junior High General	311 (21%)
	Junior High Vocational	10 (1%)
	Senior High General	187 (13%)
	Senior High Vocational	192 (13%)
	College	75 (5%)
	University (Bachelor/Master/Dr)	171 (12%)
Household composition	Household size	3.74 (1.89)
Housing characteristics	Rural	1201 (53%)
	Number of rooms ^4^	5.44 (2.53)
	House is self-owned	1575 (70%)
	Uses electricity ^4^	1969 (87%)
Flooring	Ceramic/marble/granite/stone or tiles or cement/bricks or lumber/board ^4^	1579 (70%)
Walls	Masonry (cement/prefabricated bricks) or lumber/board/plywood ^4^	1973 (87%)
Roof	Concrete or roof tiles/shingles ^4^	1947 (86%)
Drinking/cooking water	Improved source (piped, well, spring, rain)	1883 (84%)
Bath/laundry water	Improved source (piped, well, spring, rain) ^4^	2017 (89%)
Toilet type	Improved facility (own toilet, septic system) ^4^	1392 (62%)
Sewage disposal	Improved system (drainage ditch, permanent pit) ^4^	1536 (68%)
Garbage disposal	Collection by sanitation service ^4^	549 (24%)
Assets (ownership by any household member)	House and land occupied by household	1596 (71%)
	Other house or building (including land) ^4^	151 (7%)
	Vehicles (cars, boats, bicycles, motorbikes) ^4^	1168 (52%)
	Household appliances (radio, fridge, TV, etc.) ^4^	1775 (79%)
	Jewelry ^4^	1236 (55%)
	Savings ^4^	684 (30%)
	Receivables ^4^	285 (13%)
Access to credit	Know of a place to borrow money ^3^	1236 (88%)
	Ever borrowed money ^3^	42 (27%)
	Secured a loan (past 12 months) ^3^	275 (22%)
Household assistance	Received money from a community group ^3^	6 (2%)
	Received cash assistance ^3^	149 (12%)
	Received any assistance from government or NGO ^3^	455 (36%)
Expenditure	Monthly household expenditure	1,211,335.75 (1,756,339.13)
Interviewer observation of house	Moderate size yard ^4^	1344 (59%)
	Well-kept yard ^4^	1735 (77%)
	Adequate ventillation ^4^	1762 (78%)
	Stable under/next to house	447 (20%)
	House surrounded by puddles	148 (7%)
	Piles of trash around house	159 (7%)
	Human and animal waste near house	133 (6%)
Wealth index	Score	−0.28 (2.01)
	Lowest quintile	580 (26%)
	Mid-low quintile	429 (19%)
	Medium quintile	418 (19%)
	Mid-high quintile	395 (18%)
	Highest quintile	424 (19%)
Community/village characteristics ^2^	Coastal	132 (10%)
	Blocked water duct or obstructed river/gutter	320 (23%)
	Standing pools of water (not marshes, lakes)	329 (24%)

^1^ Statistics presented are mean (SD) or *n* (%). Less than 5% missing for all variables unless otherwise indicated. The first wave could be any of the 5 waves since the survey sampled new households that formed from a target member of an original household from wave 1. ^2^ >25% missing. Variables not included in modeling. ^3^ Variable not collected across all waves. Not included in full models. ^4^ Variable included in wealth index derived using principal component analysis, excluding household assets and characteristics with low factor loadings or related in the opposite direction.

**Figure 2:**
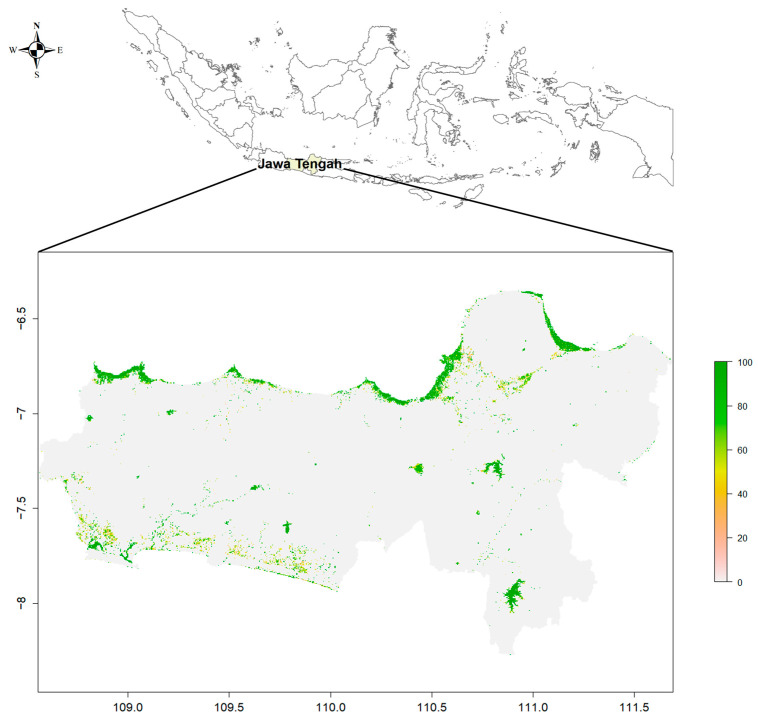
Map of Central Java showing Global Surface Water Recurrence Values 1984–2015. Recurrence values range from 0 to 100, where 0 is not water, 1 is 1% recurrence over the period, and 100 is 100% recurrence (seasonal or permanent water bodies).

Recurrent flooding was present in most districts (79%) throughout the period, with 80% of households living in affected districts. On average, small portions of the districts were affected (median percentage of district raster cells with episodic flooding = 0.5%). The most heavily affected areas in the top decile had larger portions of their districts affected (between 10 and 31%). Districts with the most recurrent flooding were Kaliwungu, Kedung, Juwana, and Pekalongan Utara. All of these were coastal districts on the north side of Central Java. Figure 2 shows recurrence values plotted on a map of Central Java. The distribution among households in our sample was highly right-skewed (skewness = 2.14, kurtosis = 6.68).

Our regression models included 95% and 97% of the total sample for food expenditure share and dietary diversity score, respectively (Table 2). We did not detect any effects between recurrent flooding and either of the outcomes. Wealth, access to credit, and home ownership were associated with improvements in both outcomes. Livestock ownership, household size, and living in a rural area were associated with worsening food expenditure share but higher diet diversity (Table 2).

When we examined recurrent flooding jointly with measures of adaptive capacity, we found associations with wealth and access to credit. Wealth was associated with improved food expenditure share and this effect was strengthened in households in more flood-prone districts. In contrast, access to credit was associated with higher odds of a more diverse diet but the direction of the effect changed to reduced odds in more flood-prone districts (Table 2).

**Figure 3:**
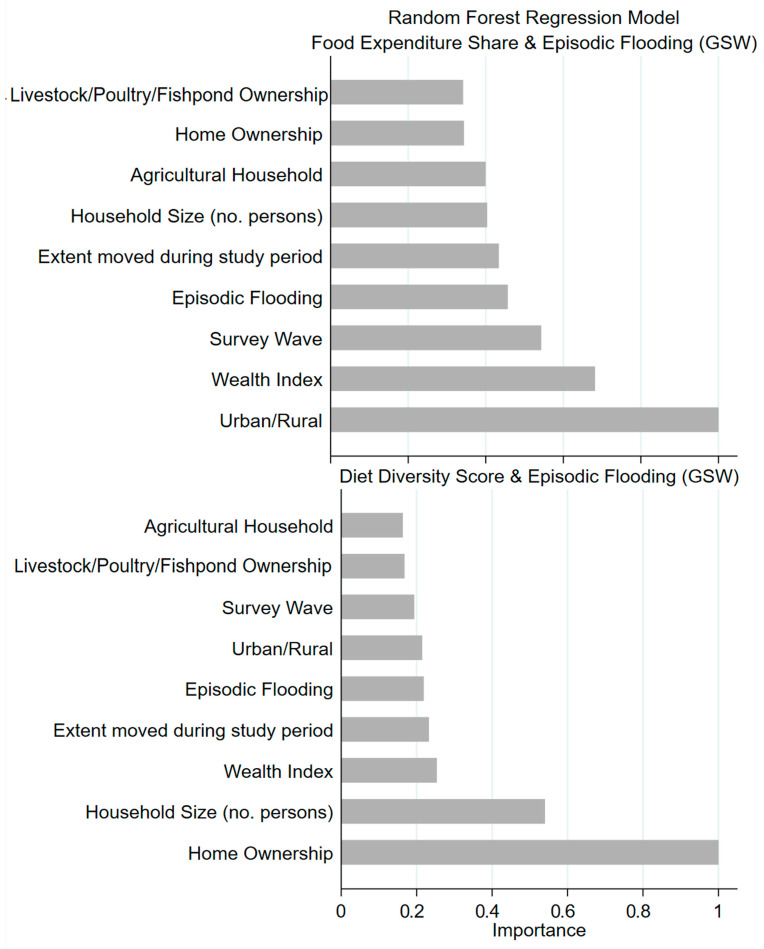
Variable importance plots.

In random forest models, flooding had more predictive importance for food expenditure share than for diet diversity in relation to the other variables (Figure 3).

## 4. Discussion

In this study, we explored a measure of flood recurrence to evaluate its impact on household diet-related aspects of food security over time in Central Java. Our flood variable measured the portions of districts affected by episodic flooding during the period (1984–2015). Our findings indicate that wealthier households in more flood-affected districts may have an advantage in terms of their ability to purchase food. Access to credit, however, which is independently associated with improved diet diversity, showed reduced diet diversity in more flood-affected districts. This could be because resources are diverted elsewhere in these areas.

We did not detect a linear, independent effect between recurring flooding and either of the outcomes. This could be due to measurement challenges related to the aggregation and scale of the variable or because the relationship has a different form and shape. We aggregated all GSW recurrence values from 0 to 99, as we did not have a prior basis from which to measure or define this variable. We attempted log transforming this variable to see if it improved linearity but it did not appear to. Since there were no substantive changes in conclusions drawn, we presented the untransformed form for interpretability. The scale of the variable was at the district rather than household level. A future direction could be to explore disaggregation of this variable further. More work is needed to compare GSW data to other flood databases and validate these measures. Nevertheless, high-resolution data like GSW are promising and provide a good-quality source to link to surveys versus other data that must be downscaled. In the absence of a universal metric for recurrent flooding or flood severity, more exploration of these data sources is important.

We explored other data products to measure flooding, including the Dartmouth Flood Observatory, which we successfully linked to the survey. However, these data were very coarse, cutting across multiple districts, and had to be aggregated down to the district level. This is problematic for analysis as there are likely large differences across districts in terms of flooded areas, which may not be accurately depicted with such a variable. Using this variable at a larger scale may be more informative. The Global Flood Awareness System is planning to introduce a flood recurrence layer in a future update. Such products will provide an important advantage over flood occurrences to understand impacts over time.

Previous efforts to measure recurrent flooding have varied, including direct interviews of affected households or indirect comparisons of affected and unaffected communities or linkages of household and spatial data [6,13,25,41]. But, while whole communities may be affected, individual households may be impacted differently. Identification of flood-affected households remains a measurement challenge. Direct questioning of households about how they are impacted by floods provides advantages and disadvantages. This information, however, is lacking over a long period of time. Thus, spatial and survey linkages provide the best opportunity to study these impacts, but these linkages are challenging. We presented and demonstrated a method for linking raster data to a household survey, but more disaggregated disaster data are needed [1].

In other similar flood-prone contexts, a common coping or adaptation strategy in response to flooding is to borrow money [8,19]. We found this to be associated with reduced odds of improved dietary diversity. Evidence from Central Java suggests that people are staying in flood-prone areas for various reasons [25,27,42]. During flood events, people are often unable to work and cook [42]. We found that wealthier households in flood-prone districts had improved food expenditure share. Taken together, these findings suggest that households that are already vulnerable may be even more disadvantaged in terms of food access when faced with flooding, while wealthier households may be better able to adapt. This may be because resources are diverted in response to flooding.

There were some data quality issues with the food access variables using the household-level data. The reference period for the food recalls used to create household dietary diversity score was 1 week, which may have led to less accurate recall than a shorter period (typically 24 h). The recall did not include a question about whether consumption during the period was normal. We attempted creating other household-level metrics, including household average dietary energy acquisition or consumption, food consumption score, household adequacy of fruit and vegetable consumption, household share of animal protein in total protein consumption, household share of dietary energy from macronutrients, and household share of energy consumed from non-staples. However, these variables could not be constructed due to lack of granularity with the food list and because the survey did not capture frequency of consumption. Some variables were not included in our models due to high missingness, including education level and community level variables, which could have been important predictors. Thus, we could not account for some of the localized factors that may also affect food security. We wanted to include a variable of whether the community had a disaster risk management plan but did not have the data. We aggregated the flood values, which could have masked the relationships in our data. Use of these data for this purpose should be further explored.

The major strength of this study was the integration of climate data to a household survey. By integrating these data streams with decades of information, we unlocked the potential to assess a host of questions which were previously not possible about the human response to the changing environment. As climate data products improve, they will be better aligned to surveys, improving our capability of conducting interdisciplinary research needed to inform policy. This study took a hybrid, blended approach between predictive and explanatory modeling to leverage the strengths of both types of modeling and glean more from the data for future insights [43].

## 5. Conclusions

We demonstrated how high-resolution data about the environment and survey data about the population can be linked into one dataset, creating new possibilities for research. We measured gradual increases in flooding throughout a 30-year span. Our findings indicate that household access to credit, a factor that ordinarily improves food access, may not be effective in flood-prone areas. Wealthier households may be better able to adapt in terms of food access. These relationships are far more complex and require further investigation. We do not yet know the impact of local policies and community support mechanisms. This is a direction for future studies. Incorporating land use data is an important next step to understand how different locales are affected. Many of the limitations in this study could be addressed with improvements in the data. As a future direction, climate data products could improve their spatiotemporal alignment with survey data. Future surveys could incorporate modules that capture households’ experiences with disasters to better identify affected households and include other economic, political, and physical factors that influence food security. Another future direction is understanding why households may be accessing credit or securing loans in flood-prone areas. Lastly, future research should focus on the repeated exposures and the cascading effects of disasters. A deeper understanding of these relationships will guide long-term climate change resilience planning.

## Figures and Tables

**Table 2:** Mixed-effect regression models examining the relationship between recurrent flooding and household food access ^1^.

	Food Expenditure Share (% of Total Spending)			Dietary Diversity Score (0 to 12)		
	**Coef.**	**SE**	***p*-Value**	**OR**	**SE**	***p*-Value**
Full model						
Recurrent flooding measure						
Fraction of district with episodic flooding (1984–2015) ^2^	5.18	7.11	0.467	1.30	1.57	0.830
Household characteristics						
Anyone was a farmer in the past year	0.60	0.49	0.223	1.10	0.08	0.187
Owns livestock/poultry/fishpond	1.06	0.45	0.020	1.29	0.08	<0.001
Household size	0.25	0.12	0.032	1.32	0.02	<0.001
Rural	2.60	0.72	0.000	1.35	0.16	0.011
House is self-owned	−1.12	0.58	0.055	1.89	0.16	<0.001
Wealth index ^3^	−2.89	0.16	0.000	1.27	0.03	<0.001
Wealth index (quadratic effect)	−0.42	0.05	0.000			
Wealth index (joint effect with fraction of district flooded episodically)	−4.56	2.17	0.035			
Extent household moved during entire study period						
Did not move	Ref.			Ref.		
Moved within the same village	−0.43	0.75	0.570	1.09	0.13	0.503
Moved within the same district	0.96	0.97	0.324	1.24	0.20	0.178
Moved within the same regency	−0.98	0.87	0.257	0.88	0.13	0.372
Moved within the same province	−1.48	0.92	0.106	0.44	0.07	<0.001
Moved to another province	−2.23	0.97	0.022	0.86	0.14	0.345
Survey wave						
Wave 1—1993	Ref.			Ref.		
Wave 2—1997	0.11	0.70	0.875	1.34	0.13	0.002
Wave 3—2000	4.11	0.69	0.000	1.94	0.19	<0.001
Wave 4—2007	2.31	0.70	0.001	1.36	0.13	0.001
Wave 5—2014	−1.85	0.71	0.009	1.05	0.10	0.630
Models examining indicators of adaptive capacity (Waves 3 to 5 only) ^4^						
Model with linear effects only						
Know of a place to borrow money	−1.83	0.81	0.023	1.47	0.18	0.001
Secured a loan in past 12 months	−0.63	0.52	0.226	1.45	0.11	<0.001
Received any assistance from government or non-governmental organization in past 12 months ^5^	1.58	0.64	0.014	1.33	0.13	0.003
Received cash assistance	−0.19	0.63	0.767	0.77	0.07	0.006
Models with differential/joint effects with flooding						
Know where to borrow and fraction of district flooded episodically	−5.84	11.80	0.620	0.01	0.02	0.010
Secured a loan and fraction of district flooded episodically	−1.03	8.57	0.904	0.15	0.20	0.147
Received any assistance and fraction of district flooded episodically	−1.29	7.39	0.861	4.16	4.48	0.184
Received cash assistance and fraction of district flooded episodically	8.77	9.22	0.342	3.10	4.14	0.398

^1^ Complete case analysis, *n* = 6412 (95% of total sample) for food expenditure share and *n* = 6513 (97% of total sample) for dietary diversity score. Linear and ordered logistic mixed-effect models for food expenditure share and dietary diversity score, respectively. ^2^ Calculated from Global Surface Water Recurrence values. Describes fraction of district raster cells with recurrence values between 1 and 99. ^3^ Joint effects not included in the Dietary Diversity Score model due to nonsignificant likelihood ratio tests. ^4^ These indicators were only available in IFLS Waves 3 to 5. Five separate models were fit, adjusted for household characteristics and survey wave: 1. model with linear effects only; 2. model with episodic flooding, knowing where to borrow, and their joint effect; 3. model with episodic flooding, securing a loan, and their joint effect; 4. model with episodic flooding, receiving any assistance, and their joint effect; 5. model with episodic flooding, receiving cash assistance, and their joint effect. Each of the models included complete case analysis of *n* = 4579 observations (94% of total sample in Waves 3 to 5). ^5^ In the form of money, rice, vegetables, sugar, fruit, meat, snacks, other food, cooking oil, or kerosene.

## Data Availability

The data presented in this study are available in GitHub at https://github.com/BreeLanglois/Indonesia-floods-foodsecurity. These data were derived from the following resources available in the public domain: The Indonesian Family Life Survey (https://www.rand.org/well-being/social-and-behavioral-policy/data/FLS/IFLS/access.html, accessed on 1 August 2024), and the Global Surface Water datasets (https://global-surface-water.appspot.com/download, accessed on 1 August 2024).
